# Evaluation of the expression of angiogenic factors in breast cancer after curcumin treatment

**DOI:** 10.1186/1753-6561-7-S2-P40

**Published:** 2013-04-04

**Authors:** Lívia C Ferreira, Bruna V Jardim, Thaiz F Borin, Marina G Moschetta, Gabriela B Gelaleti, Larissa B Maschio, Camila Leonel, Juliana R Lopes, Naiane N Gonçalves, Gustavo R Martins, Debora APC Zuccari

**Affiliations:** 1 Department of Molecular Biology, Postgraduate Program in Health Science/Faculdade de Medicina de São José do Rio Preto, FAMERP, São José do Rio Preto (SP) Brazil; 2 Department of Biology, Postgraduate Program in Genetics/Universidade Estadual Paulista - UNESP/IBILCE, São José do Rio Preto (SP) Brazil; 3 Department of Molecular Biology – Laboratory of Molecular Cancer Investigation, Faculdade de Medicina de São José do Rio Preto, FAMERP, São José do Rio Preto (SP) Brazil

## Background

Angiogenesis plays an important role in the pathogenesis of several malignancies, including breast cancer. Tumor growth requires the formation of new blood vessels that are stimulated by angiogenic factors, which initiates the sprouting and proliferation of endothelial cells. Curcumin is used as food and in traditional medicine, however evidences indicate that rhizome has anticancer effects against different types of cancers. We evaluated the effects of curcumin treatment on angiogenesis in breast cancer.

## Materials and methods

Breast cancer cell line MDA-MB-231 was cultured *in vitro* in DMEM high glucose at 37°C in 5% CO_2_. Cell viability was measured by MTT assay with three concentrations of curcumin (10ug, 20ug and 40ug), in 4 hours and 24 hours. *In vivo*, cells were injected subcutaneously in mammary gland in athymic nude mice, and each animal received daily 7,5mg of curcumin administred intraperitoneally. Tumor size was measured weekly and angiogenic factors were evaluated in breast tumors.

## Results

There was a significantly decrease in cells viability after treatment with curcumin (all concentrations). In addition, results showed that 40ug of curcumin was able to reduced 88% of cell viability after 24 hours. Furthermore, the action of curcumin as anti-angiogenic agent was tested in breast cancer xenografts established in nude mice.

## Conclusions

The highest dose of curcumin was considered the optimal concentration for *in vitro* treatment. This study was an innovative way to evaluate the potential effectiveness of curcumin in the control of angiogenesis in breast cancer.

## Financial support

FAPESP.

